# The Maize *Corngrass1* miRNA-Regulated Developmental Alterations Are Restored by a Bacterial ADP-Glucose Pyrophosphorylase in Transgenic Tobacco

**DOI:** 10.1155/2018/8581258

**Published:** 2018-09-26

**Authors:** Ayalew Ligaba-Osena, Kay DiMarco, Tom L. Richard, Bertrand Hankoua

**Affiliations:** ^1^College of Agriculture and Related Sciences, Delaware State University, 1200 N DuPont Highway, Dover, DE 19901, USA; ^2^2217 Earth and Engineering Sciences, Pennsylvania State University, University Park, PA 16802, USA; ^3^Agricultural and Biological Engineering, Pennsylvania State University, 132 Land and Water Research Building, PA 16802, USA

## Abstract

Crop-based bioethanol has raised concerns about competition with food and feed supplies, and technologies for second- and third-generation biofuels are still under development. Alternative feedstocks could fill this gap if they can be converted to biofuels using current sugar- or starch-to-ethanol technologies. The aim of this study was to enhance carbohydrate accumulation in transgenic *Nicotiana benthamiana* by simultaneously expressing the maize *Corngrass1* miRNA (*Cg1*) and *E. coli* ADP-glucose pyrophosphorylase (*glgC*), both of which have been reported to enhance carbohydrate accumulation *in planta*. Our findings revealed that expression of *Cg1* alone increased shoot branching, delayed flowering, reduced flower organ size, and induced loss of fertility. These changes were fully restored by coexpressing *Escherichia coli glgC*. The transcript level of miRNA156 target *SQUAMOSA promoter binding-like* (*SPL*) transcription factors was suppressed severely in *Cg1*-expressing lines as compared to the wild type. Expression of *glgC* alone or in combination with *Cg1* enhanced biomass yield and total sugar content per plant, suggesting the potential of these genes in improving economically important biofuel feedstocks. A possible mechanism of the *Cg1* phenotype is discussed. However, a more detailed study including genome-wide transcriptome and metabolic analysis is needed to determine the underlying genetic elements and pathways regulating the observed developmental and metabolic changes.

## 1. Introduction

Global energy demand is predicted to grow by 37 percent by the year 2040 [1]. During the same period, the distribution of energy demand will change dramatically, triggered by faster-growing economies and rising consumption in Asia, Africa, the Middle East, and Latin America. To meet this demand, the consumption of petroleum and other liquid fuels is projected to increase from 3.78 billion gallons per day in 2012 to 5.08 billion by 2040 [2]. However, increasing risks of environmental pollution and climate change due to production and use of fossil fuels necessitate the quest for alternative energy sources [3].

Production of biofuels and other chemicals from lignocellulosic biomass has been impeded by biomass recalcitrance (the resistance of plant cell walls to enzymatic deconstruction) largely due to the presence of highly heterogenic polymer lignin, which is a major barrier to cost-effective conversion of biomass to biofuels and useful chemicals [4]. Lignin consists of three major phenylpropanoid units, syringyl, guaiacyl, and hydroxyphenyl units, and can interlock with cellulose and hemicelluloses, limiting the accessibility of these polysaccharides to cellulase and hemicellulase enzymes, respectively [5–7]. Over a period of decades, several pretreatment technologies have been developed to break down lignin in the biomass and increase conversion efficiency [8]. However, these technologies have various limitations and are not being commercialized at the pace needed to address the short-term demand for biofuels. In this context, alternative feedstocks with enhanced carbohydrate yield that are easily converted to fuels using current technology have great potential.

Advances in genetic engineering have greatly contributed to the improvement of desirable traits including enhanced biomass yields, polysaccharide content, and modification of the cell wall composition to reduce pretreatment costs [9]. For example, an increase in starch content has been achieved in transgenic potato [10] and cassava [11] tubers overexpressing the *Escherichia coli* ADP-glucose pyrophosphorylase (AGPase or *glgC*, *EC* 2.7.7.27), which catalyzes the first dedicated and rate-limiting step in starch biosynthesis. The *glgC* gene encodes a major enzyme controlling starch biosynthesis, catalyzing the conversion of glucose 1-phosphate and ATP to ADP-glucose (ADPGlc) and inorganic pyrophosphate, with the ADPGlc subsequently used by starch synthases to incorporate glucosyl units into starch [12, 13]. A mutant form of the enzyme *Glg*CG336D (the amino acid glycine at position 336 is mutated to aspartic acid), which has less sensitivity to inhibitors and activators [10, 11], was shown to enhance tuber yield in cassava by 260 percent as compared to the nontransformed wild-type plants [11]. This is likely achieved by increasing the *Glg*C-mediated sink strength for carbohydrates, increasing overall photosynthetic rate, and reducing feedback inhibition of carbohydrate assimilation [10, 11, 14]. Many starch-metabolizing enzymes are redox-regulated [15–17]. Thioredoxins (*EC* 1.8.1.9) are oxidoreductases that mediate the thiol-disulfide exchange of Cys residues and act as a reductant of the redox-regulated enzymes involved in carbohydrate metabolism [9]. Overexpression of plastidal *Trxf* gene in transgenic tobacco has been shown to increase carbohydrate biosynthesis (starch and soluble sugars) in leaves [9, 18]. Similarly, a photorespiratory bypass via posttranslational targeting of the *E. coli* glycolate catabolic pathway (consisting glycolate dehydrogenase (*EC* 1.1.99.14) subunits E, F, and G) expressed in potato [19] has been shown to increase biomass, rate of photosynthesis, and sugars (glucose, fructose, and sucrose) and transitory starch, suggesting reduced photorespiration [20–22].

In addition to these and other coding genes that produce enzymes, small noncoding RNAs (microRNAs or miRNAs) of approximately 19–24 nucleotides in length can serve as gene regulatory factors and have the potential to improve complex traits including biomass traits [22–24]. Transgenic expression of the maize tandem miRNA *Corngrass1*, which belongs to miR156, in switchgrass (*Panicum virgatum*) has been reported to completely inhibit flowering, increase perenniality, increase starch content, and improve biomass digestibility with or without pretreatment due to reduced lignin content [25, 26].

miR156 are known to target the *SPL* transcription factors, which are involved in various physiological processes including promotion of juvenile to adult phase change (heteroblasty), reproductive transition, control of male fertility, and stress responses [27, 28]. The *Arabidopsis* genome contains 16 *SPL* genes, the majority of which are targeted by miRNA156 [27, 29]. The *SPL*s control plant development by directly regulating downstream genes [30]. Based on their conserved DNA-binding domain, the *SPL*s are grouped into five clades: *SPL3/SPL4/SPL5*, *SPL9/SPL15*, *SPL2/SPL8/SPL10/SPL11*, *SPL6*, and *SPL13A/B* [23, 29, 31]. Moreover, gene expression analysis and gain-of-function and loss-of-function studies have revealed several functionally distinct groups [28] including *SPL* genes regulating control of juvenile-to-adult vegetative transition and the vegetative-to-reproductive transition (*SPL2*, *SPL9*, *SPL10*, *SPL11*, *SPL13*, and *SPL15*) and those that have been reported to play a role in promoting floral meristem identity transition (*SPL3*, *SPL4*, and *SPL5*). One of these genes, *SPL8*, has been reported to regulate male fertility/seed set, petal trichome production, and root growth [29, 32–34].

In this study, maize *Cg1* was expressed in *N. benthamiana* with or without the *E. coli glgC* with the purpose of enhancing carbohydrate content in transgenic biomass. Our findings revealed that overexpression of *Cg1* alone significantly modulated plant growth and development including delayed flowering, reduced floral organs, and complete loss of fertility. These phenotypes were restored by coexpressing the *E. coli glgC*. Possible mechanisms of phenotypic alterations in tobacco by *Cg1* and the observed antagonistic effect of *glgC* are discussed.

## 2. Material and Methods

### 2.1. Gene Cloning and Generation of Expression Constructs

**Figure 1:**
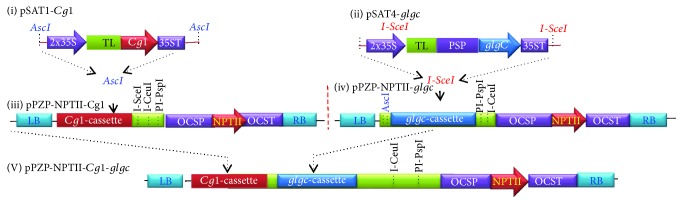
Constructs of maize *Cg1* (pSAT1-*Cg1*) (I) and *E. coli glgC* (pSAT4-*glgC*) (II) were generated in the pSAT shuttle vector under the control of enhanced 35S CaMV promoter (35S) and tobacco etch virus leader sequence (TL). The expression of *glgC* was targeted to amyloplast using pea transit peptide (PSP). Binary vectors with single and double expression cassettes pPZP-NPTII-*Cg1*, pPZP-NPTII-*glgC*, and pPZP-NPTII-*Cg1-glgC* were generated for subsequent transformation of tobacco.

The sequence of the maize *Corngrass1* (*Cg1*) which encodes two tandem miRNAs [25] (GenBank Acc. number EF541486.1) was synthesized as a gBlocks gene fragment at Integrated DNA Technologies (https://www.idtdna.com) flanked by *EcoR*I and *Kpn*I for subsequent cloning into the pSAT1 entry vector [35] under the control of the enhanced CaMV 35S promoter. The coding region of *E. coli* ADPGlc pyrophosphorylase (AGPase or *glgc*; GenBank Acc. number S58224) was amplified from the pO12 plasmid obtained from Dr. Tony Romeo (University of Florida) using sense (5′-aaggaaaggaCTCGAGatggcttctatgatatcctcttccgctgtgacaac-3′) and antisense primers, (aaggaCCCGGGgtggtgatgatgatgatgtcgctcctgtttatgccctaac) containing *Xho*I and *Sma*I, respectively. A 57-amino acid pea chloroplast transit peptide was fused to the N-terminus of the sequence to target protein expression to the amyloplast, a nonpigmented organelle responsible for starch synthesis and storage. Since single-amino acid substitution (Gly336Asp) has been shown to reduce sensitivity of the enzyme to inhibitors and activators [10], the mutation was introduced by site-directed mutagenesis using overlap extension PCR as previously described [36, 37]. *glgc* was inserted into the pSAT4 entry vector [35] also under the control of an enhanced CaMV 35S promoter. Expression cassettes of *Corngrass1* (*Cg1*) and *glgc* were assembled into the binary vector pPZP-RCS2 [38] using *Asc*I and a homing endonuclease *I-Sce*I, respectively, singly or together for coexpression. The resulting binary vectors pPZP-*NPTII*-*Cg1*, pPZP-*NPTII*-*glgc*, and pPZP-*NPTII*-*Cg1-glgc* (Figure 1) were introduced into *Agrobacterium* strain LBA4404 for subsequent transformation of tobacco (*N. benthamiana*).

### 2.2. Tobacco Transformation and Generation of Transgenic Lines

Leaf explants (~0.5 mm^2^) of 4–6-week-old tobacco were infiltrated with *Agrobacterium* harboring the expression vectors pPZP-*NPTII*-*Cg1*, pPZP-*NPTII*-*glgC*, and pPZP-*NPTII*-*Cg1-glgC* or the empty vector pPZP-NPTII for five minutes in the presence of 200 *μ*M acetosyringone. Handling of transformed tissues, selection and regeneration, and maintenance of transgenic lines were performed based on Ligaba-Osena et al. [39].

### 2.3. Validation of Transgene Insertion

To verify the insertion of transgenes, genomic DNA was isolated from 100 mg of fresh leaves of wild-type or transgenic lines using the GeneJET Plant Genomic DNA Purification Mini Kit (Thermo Scientific). The DNA was used as a template in PCR reactions to amplify the selectable marker gene (neomycin phosphotransferase, *nptII*) and *Cg1* and *glgC* genes using sense and antisense primers in Supplementary Table S1. The PCR products were analyzed by agarose gel electrophoresis.

### 2.4. RNA Extraction

Total RNA was extracted from wild-type and independent transgenic lines (*Cg1*, *Cg1*-*glgC*, or *glgC*). Fully expanded leaves of two-month-old plants were collected and immediately frozen in liquid N_2_ and ground to a fine powder using a mortar and pestle. Total RNA was isolated using Spectrum Plant Total RNA Kit (Sigma, St. Louis, USA). The RNA solution was stored at −80°C until it was used for first-strand complementary DNA synthesis.

### 2.5. Identification of Cg1 Target Genes

Given that miRNA156 has been implicated in the regulation of flowering via its downstream targets known as *SPL* transcription factors [22], we studied the expression of putative *Cg1* target genes in tobacco. By searching genome database and publications, we identified four *SPL* contiguous sequences (TC20466), EH36899 (GenBank Acc. number EH368993), and TC9706 and TC7909 [40]. EH36899 showed high homology with *SPL1*, while TC20466 is annotated as *SPL*12 (https://solgenomics.net). TC9706 (*SPL15*) and TC7909 (*SPL9*) were reported in Tang et al. [40]. To analyze the expression of target genes, primers were designed based on the contiguous sequences (Table S1). Potential *Cg1*-binding sites in the sequences of *SPL* genes were determined using the targetfinder.pl software previously developed at the Carrington lab [41].

### 2.6. Quantitative Real-Time RT-PCR

Expression of transgenes (*Cg1* and *glgC*) and putative *Cg1*-targets was studied by quantitative real-time RT-PCR (qPCR) as described previously [39]. Primers used for gene expression analysis are listed in Table S1; 18S RNA was used as an internal control. Relative expression level was calculated using the *Δ*ΔC_T_ method available on SDS software (Applied Biosystems).

### 2.7. Determination of Starch Content

The shoot biomass was ground to 1 mm particle size, and 500 mg was used for the starch assay. The biomass was preextracted to remove free sugars by incubation at 40°C water bath and filtration using Whatman 41 filter paper. The starch content in the biomass was determined according to the Dairy One procedures (Dairy One Forage Laboratory, Ithaca, NY).

### 2.8. Biomass Saccharification

To see whether coexpression of the *Cg1* and *glgC* improves saccharification efficiency, the biomass was harvested at maturity, dried in an oven at 60°C for two days, and ground to powder. One gram of ground biomass was weighed into 15 mL Falcon tubes containing 9 mL of 50 mM sodium acetate pH 5.5, and the samples were vortexed for 2 min and then centrifuged at 9000 *g* for 10 min. The supernatant was recovered to determine initial sugar content, and the pellet was washed twice with 50 mM sodium acetate buffer and centrifuged again. The final pellet was suspended in 9 mL of the 50 mM sodium acetate buffer to which 50 *μ*L of each *Trichoderma reesei* cellulase, *Aspergillus niger* glucosidase, and *Bacillus licheniformis* a-amylase (Sigma) [42] was added, with 100 *μ*L of sodium azide (from 2% stock) added to suppress microbial growth [43]. The reactions were then incubated at 45°C for three days while shaking at 250 rpm. After three days, the samples were centrifuged at 10,000 *g* for 10 min and the resulting supernatant hydrolysate was filtered (0.22 *μ*m). This hydrolysate was subsequently analyzed to determine the total reducing sugar yield or the specific sugar species released from the biomass.

### 2.9. Sugar Quantification

Sugars in the hydrolysate obtained after biomass saccharification were characterized filtered (0.22 *μ*m) for quantifying sugars using Dionex Ion Exchange Chromatography 3000 equipped with an electrochemical detector (Dionex, Sunnyvale, USA) as previously described [39]. The sugar concentration from the IC reading was converted to milligrams of sugar per gram of dry matter or milligrams of sugar per plant. Sugar yield was measured from four replicates for each treatment.

### 2.10. Statistical Analysis

Experiments were conducted in a complete randomized design in at least three replicates, and each experiment was repeated twice. Data were analyzed using one-way ANOVA using the PROC GLM procedure [44]. After the significant *F*-tests, the Tukey multiple comparison procedure was used to separate the means (*P* < 0.05).

## 3. Results

### 3.1. Modulation of Vegetative Growth by Cg1

**Figure 2:**
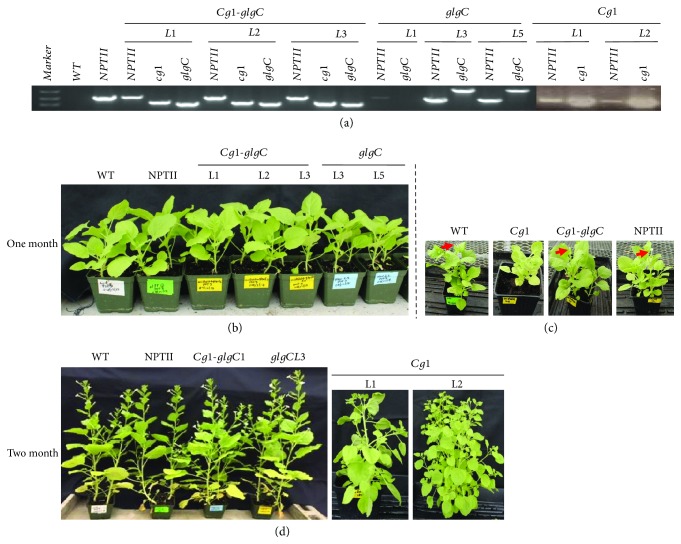
Transgenic expression of *Cg1* and *glgC* in tobacco. (a) Integration of *Cg1* and *glgC* into the tobacco genome as confirmed by PCR using genomic DNA as template. Primers specific to *NPTII*, *Cg1*, or *glgC* were used. (b) Phenotypes of transgenic (T1) and nontransformed tobacco lines after one month. (c) Phenotypes of transgenic (T0) lines regenerated from transgenic callus and nontransformed tobacco lines after one month. Arrows indicate initiation of flowering in WT, *NPTII*, and *Cg1-glgC* lines. (d) Phenotypes of transgenic (T1) and nontransformed tobacco lines obtained from seeds, and *Cg1* transgenic (T0) lines regenerated from transgenic callus (right panel) after two months of growth in the soil. Phenotypic alteration is observed in *Cg1*-expressing lines alone.

Ectopic expression of *Cg1* has been shown to alter growth and development in various plant species [25]. In this study, we investigated whether *Cg1* expressed in tobacco alone or with *glgC* affects plant growth and development. At least six independent lines were generated for each construct. Transgene insertion of at least two independent transgenic lines was validated by PCR using genomic DNA as template (Figure 2(a)).

After one month of growth, transgenic (T_1_) lines coexpressing *Cg1* and *glgC* (*Cg1-glgC*) and *glgC* were not different from the empty vector and nontransgenic control lines (Figure 2(b)). Because *Cg1* lines did not produce seeds, we compared regenerated (T_0_) *Cg1* plants with the WT, the empty vector control (NPTII), and *Cg1-glgC*. Interestingly, *Cg1* lines exhibited a distinct phenotype. The *Cg1* plants develop smaller leaves and grow slower than the controls and *Cg1-glgC* (Figure 2(c)). Likewise, in two-month-old plants (Figure 2(d)), transgenic lines expressing *Cg1-glgC* and *glgC* were not different from the nontransgenic (WT) and empty vector control lines, suggesting that coexpression of *Cg1* and *glgC* or *glgC* alone may not interfere with plant growth and development. The *Cg1* lines exhibited a bushy phenotype with increased branching and leaf number and reduced leaf size as compared to the WT and the other transgenic lines, which is more evident in two-month-old plants (Figure 2(c)) as compared to the one-month-old seedling, and is more pronounced in *Cg1*L2 (Figure 2(d)).

### 3.2. Cg1-Altered Flower Development Is Restored by glgC

**Figure 3:**
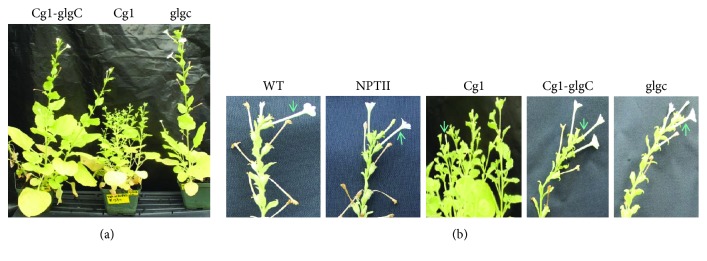
Comparison of T_0_ transgenic lines after flowering. (a) *Cg1*-expressing lines appeared more bushy than did lines expressing *glgC* or coexpressing *Cg1* and *glgC*. (b) Close-up pictures to compare floral organs between transgenic and nontransgenic wild-type and empty vector control.

In this study, expression of *Cg1* singly delayed flowering (Figures 2(c) and 3). Moreover, because the *Cg1* plants have more branching, the number of flowers per plants was higher than in the WT, and floral parts were reduced in size when visually observed (Figure 3). The flowers bore smaller petals as compared to WT, whereas there was no marked difference in flowering time (Figures 2(c) and 2(d)) and floral organ development (Figure 3) between the WT control, *Cg1-glgC*, or *glgC*. Moreover, the number of flowers per plant in *Cg1-glgC* and *glgC* does not appear to be different from that in WT. Intriguingly, none of the flowers of a total of six generated *Cg1* lines were fertile; therefore, no seed was recovered from these lines. On the contrary, the flowers of *Cg1-glgC* and *glgC* lines were fertile and produced normal seeds same as the WT or empty vector control lines. These findings suggest that coexpression of *glgC* with *Cg1* restores normal flower development and fertility.

### 3.3. Transgene Expression and Possible Regulation of Putative *N. Benthamiana* SPL Genes

Given that mR156 has been shown to modulate flowering via suppression of *SPL* transcription factors, we analyzed the transcript level of putative homologs of the *Arabidopsis SPL* genes including *SPL1*, *SPL9*, *SPL12*, and *SPL15* using quantitative PCR in WT and constitutively expressing *Cg1*, *Cg1-glgC*, or *glgC* lines. To distinguish from *Arabidopsis SPL* genes, the *SPL* genes analyzed in this study are denoted as *NbSPL* (for *N. benthamiana SPL* genes).

**Figure 4:**
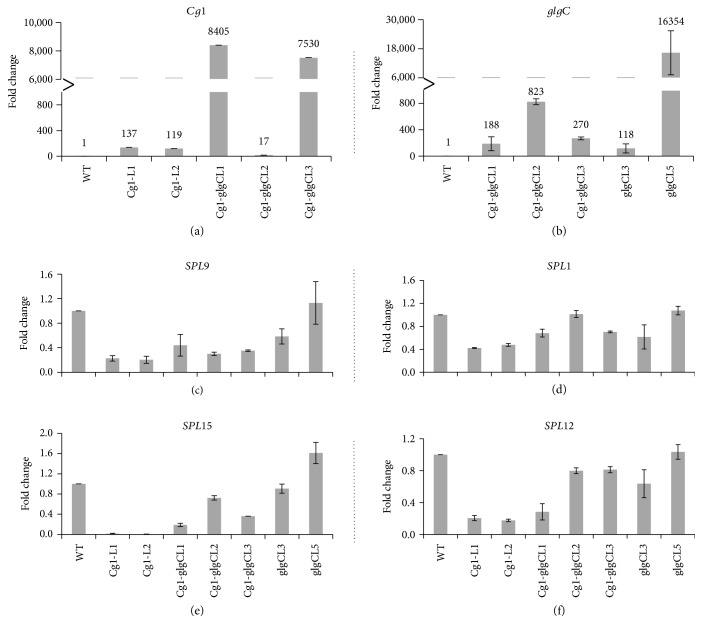
Analysis of gene expression in transgenic *N. benthamiana* using quantitative real-time PCR. First-strand cDNA was synthesized from total RNA isolated from two-month-old greenhouse established plants as described in the Material and Methods. Expression of *Cg1* (a), *glgC* (b), and putative *Cg1* targets *SPL9* (c), *SPL1* (d), *SPL15* (e), and *SPL12* (f) was determined by using transcript accumulation in the WT sample reference. Transcript level is expressed as fold change as compared to the WT. Bars represent means and standard error of four replicates. Experiments were repeated twice.

As shown in Figure 4, transcripts of both *Cg1* and *glgC* were accumulated at higher levels in the transgenic lines as compared to the nontransgenic control (WT). In *Cg1-glgC* lines, the transcript levels of *Cg1* increased by up to 8000-fold (Figure 4(a)) as compared to the WT while the level of *Cg1* transcript accumulation was over 120-fold higher than in WT. Interestingly, the transcripts of *Cg1* in *Cg1-glgC* lines were over 60-fold higher than in *Cg1* lines. Likewise, the transcripts of *GlgC* were abundantly accumulated in the *Cg1-glgC* and *glgC* lines (Figure 4(b). The increase in transcript abundance ranged from about 120- to 16,000-fold as compared to the WT. The highest increase in *glgC* transcript was detected in *glgCL5*.

On the other hand, transcript levels of all putative *NbSPL* genes analyzed were downregulated in most of the transgenic lines. This decrease in transcript levels was more severe in *Cg1* lines. Expression of *NbSPL1*, *NbSPL9*, *NbSPL12*, and *NbSPL15* was severely downregulated in the *Cg1* lines as compared to the WT, *Cg1-glgC*, or *glgC* (Figures 4(a)–4(d)). Expression of *NbSPL15* was more severely suppressed in the *Cg1* lines (Figures 4(c) and 4(e)). The expression of *NbSPL9* and *NbSPL15* was also downregulated in *Cg1-glgC*-coexpressing lines, but less severely compared to *Cg1* lines. Expression of *NbSPL1* and *NbSPL12* was not markedly affected in the *Cg1-glgC* lines (Figures 4(d) and 4(f)). Similarly, expression of the *SPL* genes in *glgC* lines was not markedly affected (Figure 4). These findings suggest that coexpression of *glgC* restores expression of *NbSPL* genes that was suppressed by *Cg1.*

### 3.4. Identification of Cg1 and Putative NbSPL Complementary Sites

Since the observed phenotype of the transgenic lines and the gene expression data suggest regulation of the *AtSPL* paralogue genes in tobacco by *Cg1*, we searched for the *Cg1*-binding sites in the putative *SPL* sequences using the targetfinder.pl software [41]. Putative *Cg1*-binding sites with high complementarity were identified in all the *SPL* genes (Supplementary Figure S1). While the complementary site was detected in the ORF of *NbSPL1*, *NbSPL9*, and *NbSPL15*, it was detected in the 3′-UTR region of *NbSPL12* as previously reported for *Arabidopsis SPL3/4/5* [45, 46].

### 3.5. Overexpression of glgC with or without Cg1 Increases Shoot Dry Matter Yield

**Figure 5:**
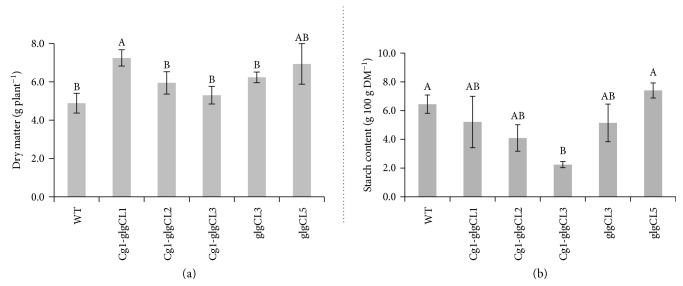
Determination of biomass and starch content. (a) Matured plants were harvested, and dry matter was determined after drying in an oven at 60°C for two days. (b) Starch content in dried biomass was determined according the Dairy One procedures as described in the Material and Methods. Bars represent means and standard error of three replicates. Experiments were repeated twice. Bars bearing the same letter are not significantly different (*P* < 0.05).

To see the effect of *glgC* on biomass accumulation, dry matter yield of *Cg1-glgC*- and *glgC*-expressing T1 lines and the WT was determined. Because the *Cg1* lines failed to produce seeds, we were not able to compare the biomass yield of *Cg1* lines with that of the WT and lines expressing *Cg1-glgC* and *glgC*. As shown in Figure 5(a), coexpressing lines *Cg1-glgCL1* and *glgCL5* showed between 9 and 48% increase in shoot biomass. In lines *Cg1-glgCL1* and *glgCL5*, biomass yield was increased by 48% and 42%, respectively, followed by *glgCL3* (28%) and *Cg1-glgCL2* (22%), while biomass yield of *Cg1-glgCL3* was only 9% higher than that of the WT.

### 3.6. Carbohydrate Content in WT and Transgenic Lines

To understand whether overexpression of *glgC* modulates carbohydrate content, we determined starch and sugar content in mature WT and transgenic lines. The analysis showed that the starch content at maturity was slightly reduced in the transgenic lines overexpressing *glgC* alone or significantly reduced in a line coexpressing *glg*c and *Cg1* as compared to the WT (Figure 5(b)). The starch content in the *Cg1-glgC*-expressing line was reduced by up to threefold. This could be due to the age at which starch content was determined. However, whether decrease in starch content is due to age-dependent changes in carbohydrate dynamics or experimental procedures needs to be determined in detail through future studies.

**Figure 6:**
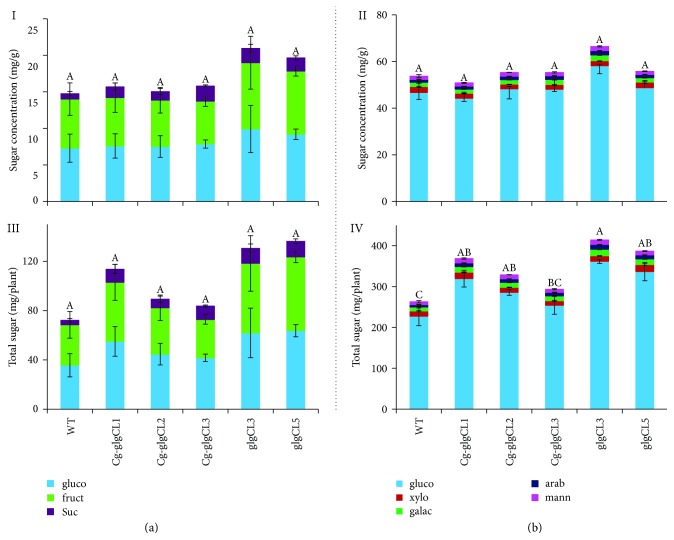
Biomass saccharification efficiency. Biomass was harvested at maturity and dried in an oven at 60°C for two days. One gram of ground biomass was used for the analysis as described in Material and Methods. Sugars released before (a) or after (b) saccharification with a cocktail of *α*-amylase, cellulase, and glucosidase were quantified using IC DIONEX. Bars represent means and standard error of four replicates, and measurement was repeated twice. Bars bearing the same letter are not significantly different (*P* < 0.05).

The concentration of sugars was determined using IC-Dionex in the presence of standards of known sugar concentration. As shown in Figure 6 and Supplementary Table S2, glucose, fructose, and sucrose were the major sugars detected in the samples prior to saccharification by a cocktail of *α*-amylase, cellulase, and glucosidase. As compared to the WT, slightly more fructose and sucrose were released from the *glgC* lines (Figure 6(a), I). The amount of sugars released from *Cg1-*glgC lines was not different from that in the WT. However, the sugar content calculated based on total dry matter per plant in transgenic lines (*Cg1-glgCL1*, *GlgCL3*, and *glgCL5*) was slightly higher than in the WT (Figure 6(a), II) because these lines produced more biomass. Wild-type and transgenic biomasses were subjected to saccharification by a cocktail of *α*-amylase, cellulase, and glucosidase enzymes to release more sugars. After three days of saccharification, hexose (glucose, galactose, and mannose), and pentose (xylose and arabinose) sugars were released (Figure 6(b), Supplementary Table S3). Glucose was the dominant sugar released from all the tobacco lines (transgenic as well as control lines) (51–66 mg/g DM) accounting for 87% of the total sugars released. The release of glucose was enhanced by saccharification by about fivefold. There was no marked difference in the amount of sugars released from most of the transgenic lines and the WT control, while only *glgC*L3 released slightly more sugars (66 mg/g DM) than the WT did (51 mg/g DM), as well as the rest of the transgenic lines (Figure 6(b), I). Moreover, *GlgCL3* released at least 20% more glucose than the other transgenic lines did. The amount of glucose released from the *Cg1-glgC*-expressing lines was not significantly different from that in WT plants. There was no marked difference among the lines in the amount of galactose, mannose, xylose, and arabinose released. However, the total amount of sugars (mg/plant) released from most of the transgenic lines (*Cg-glgCL1*, *GlgCL2*, *glgCL3*, and *glgCL3*) was significantly higher than in the WT control (Figure 6(b), II). Moreover, the total amount of sugars produced in lines overexpressing *glgC* was slightly higher than in WT and *Cg1-glgC* lines.

### 3.7. Discussion

Overexpression of the maize *Cg1* in various plant species has been shown to enhance sugar and starch content [25, 26]. Likewise, expression of *glgC* has been shown to increase sink strength and starch accumulation in transgenic potato [47] and cassava [11]. Therefore, this study was conceived to see whether simultaneous expression of the two genes could modulate carbohydrate metabolism in the transgenic biomass.

### 3.8. Overexpression of Cg1 Alters Vegetative Growth

In this study, the growth of *Cg1*-expressing lines was significantly altered. At the early stage, the growth of *Cg1* lines was slower than that of the nontransgenic control (Figure 2(b)). Moreover, *Cg1* lines produced smaller and more leaves and branches as compared to the WT control. The observed increase in lateral growth could be due to a decrease in apical dominance [48] as reported previously in various plant species expressing *Cg1* [25, 26] or miRNA156 [49, 50]. The maize *Cg1* expressed in *Arabidopsis*, *Brachypodium*, switchgrass [25], and poplar [51] has been shown to produce plants with extra branches and leaves, while in corn, *Cg1* has been shown to increase the number of tillers [52]. Similarly, overexpression of the rice stem-loop fragment of the *OsmiR156b* precursor in switchgrass has been shown to increase tiller number [26]. Reduced leaf size and increased leaf number and alteration of other morphological traits have also been observed in tobacco (*N. tabacum*) overexpressing the *Arabidopsis* miR156A hairpin structure [50].

As reported by Muller and Leyser [53], variation in shoot architecture depends on the formation of axillary meristems and the subsequent regulation of their activation, which depends on the genotype, developmental stage, and environment, which in turn is mediated by hormonal signals. Axillary bud outgrowth and branching are mainly controlled by apical dominance and the crosstalk between plant hormones auxin, cytokinin, and strigolactone [53–55]. In this study, the *Cg1* lines exhibited a bushy phenotype with decreased plant height and increased branching (Figures 2 and 3). This suggests that *Cg1* may affect hormone balance, for example, decreasing the level of auxin and strigolactone or increasing the level of cytokinin; however, this remains to be studied.

### 3.9. Overexpression of Cg1 Alters Reproductive Development

In this study, we observed a delay in the transition from vegetative to reproductive phase in *Cg1* lines. As compared to the WT and transgenic lines (*NPTII*, *Cg1-glgC*, and *glgC*), flower initiation was delayed in *Cg1* lines. Initiation of flowering was observed in less than two months in the former while it was delayed for about two weeks in the latter (Figures 2(c) and 2(d)). This observation is consistent with previous reports on the prolonged juvenile phase in various plant species expressing *miRNAs* [48, 49, 51, 52].

It is well-established that members of the miRNA miR156 have been shown to prolong juvenile cell identities and delay flowering by targeting the transcripts of the *SPL* transcription factors, which in turn activate the expression of flowering regulators such as *LEAFY* and *APETALA1* [56] and a different microRNA, *miR172* [57]. Overexpression of *Cg1* in switchgrass has been shown to downregulate the expression of four *SPL* homologs [25] as compared to the nontransgenic control. In this study, we analyzed the gene expression levels of *NbSPL1*, *NbSPL9*, *NbSPL12*, and *NbSPL15* (Figure 4). Expression of the *NbSPL* genes was more severely suppressed in *Cg1*- and *Cg1-glgC*-expressing lines as compared to the WT and *glgC* lines. The reduction in transcript level was more severe in *Cg1*, particularly for *NbSPL15*, which was downregulated by nearly a hundred fold, whereas the expression of closely related paralogue *NbSPL9* is suppressed by fivefold. In *Arabidopsis*, both *SPL9* and *SPL15* have been shown to redundantly regulate juvenile to adult phase transition [58]. *AtSPL15* has been implicated in the coordination of basal floral promotion pathways required for flowering in noninductive environments. *AtSPL15* has been shown to directly activate transcription of the MADS-box floral activator *FRUITFULL* (FUL) and *miR172b* in the shoot apical meristem and during floral induction, whereas *AtSPL9* is expressed later in flanks of the shoot apical meristem [59] and has also been implicated in the regulation of *miR172b* [60]. In contrast to miR156 which delays flowering by suppressing the expression of *SPL*s, *miR172* activates flowering by facilitating the degradation of its target transcription factors related to the *APETALA2* (AP2) gene, including *TARGET OF EAT1* (*TOE1*), *TOE2*, *TOE3*, *SCHLAFMUTZE* (*SMZ*), and *SCHNARCHZAPFEN* (*SNZ*), which are implicated in repressing the floral inducer *Flowering Locus T* (FT) [61], whereas SPL9 has been shown to induce flowering through activating MADS-box genes APETALA1 (AP1), FRUITFULL (FUL), and SUPPRESSOR OF OVEREXPRESSION OF CO1 (SOC1) [56, 62].

The expression level of *NbSPL1* and *NbSPL12* is also suppressed in *Cg1*-expressing lines as compared to the WT (Figures 4(d) and 4(f)), suggesting the presence of interaction between *Cg1* and *NbSPL1/SPL12* while the transcript level was not markedly affected in *Cg1-glgC* and *glgC* lines. *AtSPL1*, *AtSPL12*, and *AtSPL14* are expressed most strongly in cauline leaves (growing on the upper part of the stem), flowers, and latest-age shoot apices [63]. The role of *SPL1* and *SPL12*, both lacking negative regulation by *miR156* and *miR157* [2], in flowering is not well understood. However, recent reports suggest that *SPL1* and *SPL12* control the expression of many genes and regulate multiple biological processes in *Arabidopsis* inflorescence upon heat stress [30]. Given that miRNA156 has been implicated in various developmental processes including flowering time, flower fertility, alteration of cell wall composition, and biotic and abiotic stress responses [64], downregulation of *NbSPL1* and *NbSPL12* homologs in this study may suggest the presence of developmental processes regulated by the interaction of *Cg1-NbSPL1/SPL12* which needs to be identified in the future.

### 3.10. Overexpression of Cg1 Induces Flower Sterility

Seed production is a key step in the survival of flowering plants; however, its success depends on favorable genetic and environmental factors supporting optimum flower development and fertility. Our findings revealed that flowers of the *Cg1*-expressing lines were fully sterile, and no seed was recovered from these lines. Although the mechanism of observed sterility in tobacco expressing *Cg1* is yet to be understood, it is likely that flower fertility is regulated by *Cg1* and its target *SPL* paralogue genes as previously reported. In *Arabidopsis*, fully fertile flowers require the action of *AtSPL8* which functions redundantly with multiple miR156/7-targeted SPL genes including *AtSPL2*, *AtSPL9*, and *AtSPL15* [33]. In the current study, flower development and fertility were not affected in *Cg1-glgC*-coexpressing lines (Figures 2 and 3). The *Cg1-glgC* lines were fully fertile and produced normal seeds, which suggests that a pathway regulated by coexpression of *glgC* restores floral fertility that was suppressed by *Cg1. AtSPL8* is required for proper development of sporogenic tissues in *Arabidopsis* as early anther development has been shown to be affected in *AtSPL8* mutants (*spl8-1*) overexpressing miR156b, resulting in the development of small and fully sterile anthers [33]. Furthermore, *AtSPL8* and miR156-targeted *SPL* genes also regulate gynoecium development by interfering with auxin homeostasis and signaling [34]. Therefore, the absence of seed formation in *Cg1*-expressing lines could be due to suppression of a yet to be identified *AtSPL8* paralogue in tobacco. While it hampers plant fecundity, *Cg1*-induced pollen sterility has a significant biotechnological implication, for example, in eliminating the risk of transgene escape, which is one of the major concerns associated with GM crops [25].

### 3.11. Possible Role of Carbohydrates in Flower Initiation and Fertility

Our findings revealed that coexpression of *Cg1* and *glgC* does not have a marked effect on flowering time and flower fertility. The phenotype of *Cg1-glgC* was not different from the nontransgenic control, despite the up to sixtyfold higher *Cg1* transcript level in *Cg1-glgC* lines as compared to those expressing *Cg1* alone (Figure 4(b)). This suggests that besides *Cg1(miR156)-SPL* interaction, there is likely a *glgC-*sensitive pathway that is involved in the regulation of various aspects of flowering including flower initiation, flower organ development, and fertility. Therefore, since AGPase is a major enzyme controlling starch biosynthesis [13], reconstitution of normal flowering in *Cg1-glgC* lines as compared to *Cg1* lines could be due to *glgC*-mediated metabolic changes. Involvement of carbohydrates in developmental transitions has been reported before [64–69].

Trehalose-6-phosphate (T6P), which is an indicator of carbohydrate status in plants, has been implicated in the regulation of flowering [65]. A reduction in Tre6P by the loss-of-function mutation of TREHALOSE-6-PHOSPHATE SYNTHASE 1 (*TPS1*), an enzyme which converts UDP-glucose to Tre6P, has been shown to delay flowering in *Arabidopsis*, even under flower inductive environmental conditions [65, 70]. Wahl et al. [66] further showed that the Tre6P pathway controls expression of *SPL* genes via or independently of miR156. Tre6P has also been shown to regulate starch metabolism in plants [71–73]. For example, exogenous application of trehalose in *Arabidopsis* induced accumulation of starch by increasing the activity of AGPase [71], which has been suggested to be via a thioredoxin-mediated redox reaction [74]. Furthermore, rising sucrose levels in plants are accompanied by increases in the level of Tre6P, redox activation of AGPase, and stimulation of starch synthesis *in vivo* [71]. Although it remains to be studied, the lack of an abnormal phenotype in *glgC*-expressing tobacco lines in the current study could be due to Tre6P-mediated stimulation of AGPase activity and starch synthesis and alteration of carbohydrate dynamics during flower initiation and early floral development. Various genetic and physiological approaches have demonstrated the involvement of starch in the control of floral induction [75] and fertility [76–78].

### 3.12. Overexpression of glgC Increases Biomass and Sugar Yield

The biomass yield of transgenic lines expressing *Cg1-glgC* and *glgC* was higher than in the WT control. This increase in yield is likely due to *glgC. glgC* has been shown to increase biomass yield in cassava [11] and potato [46] by increasing sink strength for assimilates and releasing possible feedback inhibition on overall photosynthesis or carbon fixation [11]. On the contrary, starch content was not enhanced in *glgC*-expressing lines (Figure 5) as determined from biomass that was harvested after physiological maturity, which could be due to plant age. Therefore, a more detailed study is needed to determine the level of starch and sugars at different developmental stages. The amount of sugars released from the *Cg1-glgC* lines before and after saccharification was not different from the WT control. On the other hand, the amount of sugars released from *glgC* lines was slightly higher than in the WT (Figures 6(a-I) and 6(b-II)). However, the total sugar yield per plant was higher in both *Cg1-glgC* and *glgC* lines (Figures 6(a-III) and 6(b-IV)), suggesting that overexpression of *glgC* with or without *Cg1* has a potential to increase overall sugar production as biofuel feedstocks. Taken together, overexpression of *glgC* only increased total sugar production and sugar release while this was not observed in *Cg1-glgC* lines. Enhanced total sugar content per plant was observed for *glgC* and *Cg1-glgC* expressers as compared to the nontransgenic control, suggesting a potential of the transgenic approach to increase sugar production.

## 4. Conclusion

**Figure 7:**
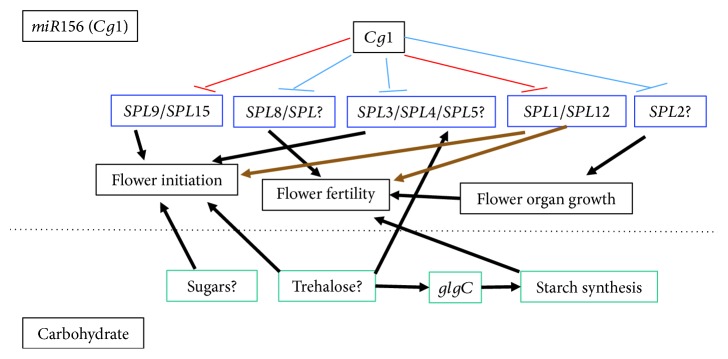
A simplified model proposing possible *miR156 (Cg1*) and carbohydrate-mediated pathways regulating flowering traits in *Cg1*- and *glgC*-expressing transgenic tobacco. Red lines, genes downregulated by *Cg1* in this study. Blue lines, miR156 target genes reported elsewhere and discussed here. Brown arrows, roles not yet reported. Black arrows, roles reported elsewhere and discussed here.

*Cg1* expression in transgenic tobacco altered vegetative growth, delayed flowering, and led to loss of fertility. Coexpression of *glgC* with *Cg1* restored wild-type phenotype. *Cg1*-induced changes in vegetative and reproductive growth are likely regulated via suppression of its target *SPL* genes. The antagonistic effect of *glgC* in restoring the *Cg1* phenotype may suggest involvement of changes in carbohydrate dynamics in flower initiation and fertility. Based on the gene expression analysis and reviewed literature, we propose a model summarizing how *Cg1* could modulate flowering and fertility by downregulating the expression of its target *SPL*s, as well as possible involvement of carbohydrates in flower initiation and fertility (Figure 7). Overexpression of *Cg1* leads to downregulation of *SPL* transcription factors, which in turn regulates flower initiation, organ growth, and fertility. Likewise, carbohydrates including sugars, trehalose, and AGPase-mediated enhanced starch biosynthesis may be involved in the regulation of flower development and fertility. Future studies will focus on deciphering genetic and physiological mechanisms regulating the observed phenotype. Global transcriptome and metabolomic analysis as well as biomass composition analysis will help in identifying key genetic elements and pathways regulating the observed phenotypes.

## Data Availability

All relevant data are available from the corresponding authors upon request.

## Supplementary Materials

Supplementary Materials Supplementary Table S1: primers used for validation of transgenic lines and for gene expression analysis. Supplementary Table S2: composition of free carbohydrates prior to biomass saccharification. Supplementary Table S3: composition of free carbohydrates after biomass saccharification. Supplementary Figure S1: identification of Cg1-binding sites in SPLs.
