# Supplementary material for: The Maize Corngrass1 miRNA-Regulated Developmental Alterations Are Restored by a Bacterial ADP-Glucose Pyrophosphorylase in Transgenic Tobacco
Source: Int J Genomics. 2018 Sep 26;2018:8581258. doi: 10.1155/2018/8581258 (PMC6178181; doi:10.1155/2018/8581258)

Supplementary Table S1. Primers used for validation of transgenic lines (gPCR), and for gene expression analysis (qPCR) for maize *Cg1,* *E. coli* (*glgC*), putative *N. benthamiana* *Cg1* targets (TC20466, TC9706, EH36899 and TC7909), and the internal control (18S RNA).

|  | Gene | Sense primer | Antisense primer |
| --- | --- | --- | --- |
| gPCR | *NPTII* | atggggattgaacaagatggattgc | gaagaactcgtcaagaaggcgatag |
|  | *Cg1* | aaggaaagggaattcaccacccaaataagcataaatagtagtggttg | aaggaaaggggtacccatcgaacatgatagcgatcgatctctgtgtc |
|  | *glgC* | aaggaaaggactcgagatggcttctatgatatcctcttccgctgtgacaac | aaggacccgggctagtggtgatgatgatgatgtcgctcctgtttatgccctaac |
| *qPCR* | *Cg1* | catgagtgccatgctaggag | tagcgatcgatctctgtgtca |
|  | *glgC* | agaactccagccacgactttg | tacccacatcgcgccagtac |
|  | EH36899 (*SPL1*) | cttagagagatggaagataac | cttccggagagagtcaatac |
|  | TC7909 (*SPL9*) | gtcctggtattattccttcc | caccactcgacaaattgaag |
|  | TC20466 (*SPL12*) | actggggaaacctggtaatg | gtgttgaccaagccgttaac |
|  | TC9706(*SPL15*) | gttctgcaaggttcacccac | gtggattccttgatccccag |
|  | 18S RNA | cgcaagaccgaaactcaaag | tgttcatatgtcaagggctg |

Table. S2. Composition of free carbohydrates prior to saccharification as determined by ion chromatography (IC). One gram of ground biomass was suspended in 9 mL 50 mM sodium acetate (pH 5.5). The amount of sugars released in the hydrolysate was determined based on standards of known concentration for each sugar species using IC as described in the Materials and Methods section. Values are means and standard error (n = 4) (Glg = glucose, Fru = fructose and Suc = Sucrose). Means followed by the same letter are not significantly different (P < 0.05).

|  | Sugar concentration (mg/g) | | | Total sugar (mg/g) | Total sugar content (mg/Plant) | | | Total sugar  (mg/plant) |
| --- | --- | --- | --- | --- | --- | --- | --- | --- |
| Line | Glc | Fru | Suc |  | Glc | Fru | Suc |  |
| WT | 7 + 2 | 7 + 2 | 0.8 + 0 | 14.8 + 4.2a | 36 + 9 | 33 + 11 | 4 + 1 | 72.1 + 20.5a |
| Cg-glgCL1 | 8 + 2 | 7 + 2 | 1.5 + 1 | 15.7 + 4.1a | 55 + 12 | 48 + 15 | 11 + 4 | 113.7 + 29.4a |
| Cg-glgCL2 | 8 + 2 | 6 + 2 | 1.2 + 0 | 15.0 + 3.1a | 45 + 9 | 38 + 10 | 7 + 2 | 89.3 + 18.6a |
| Cg-glgCL3 | 8 + 1 | 6 + 1 | 2.1 + 0 | 15.8 + 1.3a | 42 + 3 | 31 + 4 | 11 + 1 | 83.8 + 7.0a |
| glgCL3 | 10 + 3 | 9 + 4 | 2.0 + 1 | 21.0 + 7.4a | 62 + 20 | 57 + 23 | 12 + 4 | 130.6 + 46.2a |
| glgCL5 | 9 + 1 | 9 + 1 | 1.8 + 0 | 19.8 + 1.9a | 64 + 5 | 60 + 5 | 13 + 2 | 136.2 + 13.2a |

Table. S3. Composition of sugars after saccharification using a cocktail of a-amylase, cellulase and glucosidase as determined by ion chromatography. One gram of ground biomass was suspended in 9 mL 50 mM sodium acetate (pH 5.5). The amount of sugars released in the hydrolysate was determined based on standards of known concentration for each sugar species using IC as described in the Materials and Methods section. Values are means and standard error (n = 4) (Glc = glucose, Xyl = Xylose, Gal = galactose, Ara = arabinose, Man = Mannose). Means followed by the same letter are not significantly different (P < 0.05).

| Line | Sugar concentration (mg/g) | | | | | Total sugar  (mg/g) | Total sugar content (mg/Plant) | | | | | Total sugar  (mg/plant) |
| --- | --- | --- | --- | --- | --- | --- | --- | --- | --- | --- | --- | --- |
|  | Glc | Xyl | Gal | Ara | Man |  | Glc | Xyl | Gal | Ara | Man |  |
| WT | 47 + 5 | 3 + 0 | 2 + 0 | 1 + 0 | 2 + 1 | 53.8 + 5.7a | 227 + 23 | 13 + 1 | 10 + 2 | 6 + 1 | 7 + 3 | 262.8 + 27.7c |
| Cg-glgCL1 | 44 + 3 | 2 + 0 | 2 + 0 | 1 + 0 | 2 + 0 | 50.9 + 3.1a | 319 + 20 | 15 + 1 | 14 + 1 | 9 + 1 | 11 + 2 | 368.7 + 22.3ab |
| Cg-glgCL2 | 48 + 1 | 2 + 0 | 2 + 0 | 1 + 0 | 2 + 0 | 53.3 + 1.2a | 286 + 7 | 12 + 1 | 12 + 1 | 9 + 0 | 10 + 0 | 328.7 + 7.1ab |
| Cg-glgCL3 | 48 + 4 | 2 + 0 | 2 + 0 | 2 + 0 | 2 + 0 | 53.3 + 4.7a | 254 + 22 | 11 + 1 | 12 + 1 | 8 + 1 | 8 + 2 | 293.5 + 24.8bc |
| glgCL3 | 58 + 1 | 2 + 0 | 3 + 0 | 2 + 0 | 2 + 0 | 66.5 + 0.7a | 362 + 5 | 13 + 1 | 16 + 1 | 11 + 0 | 12 + 1 | 414.3 + 4.2a |
| glgCL5 | 49 + 3 | 2 + 0 | 2 + 0 | 1 + 0 | 1 + 0 | 55.8 + 3.7a | 337 + 23 | 17 + 2 | 14 + 1 | 10 + 1 | 10 + 1 | 387.0 + 25.7ab |


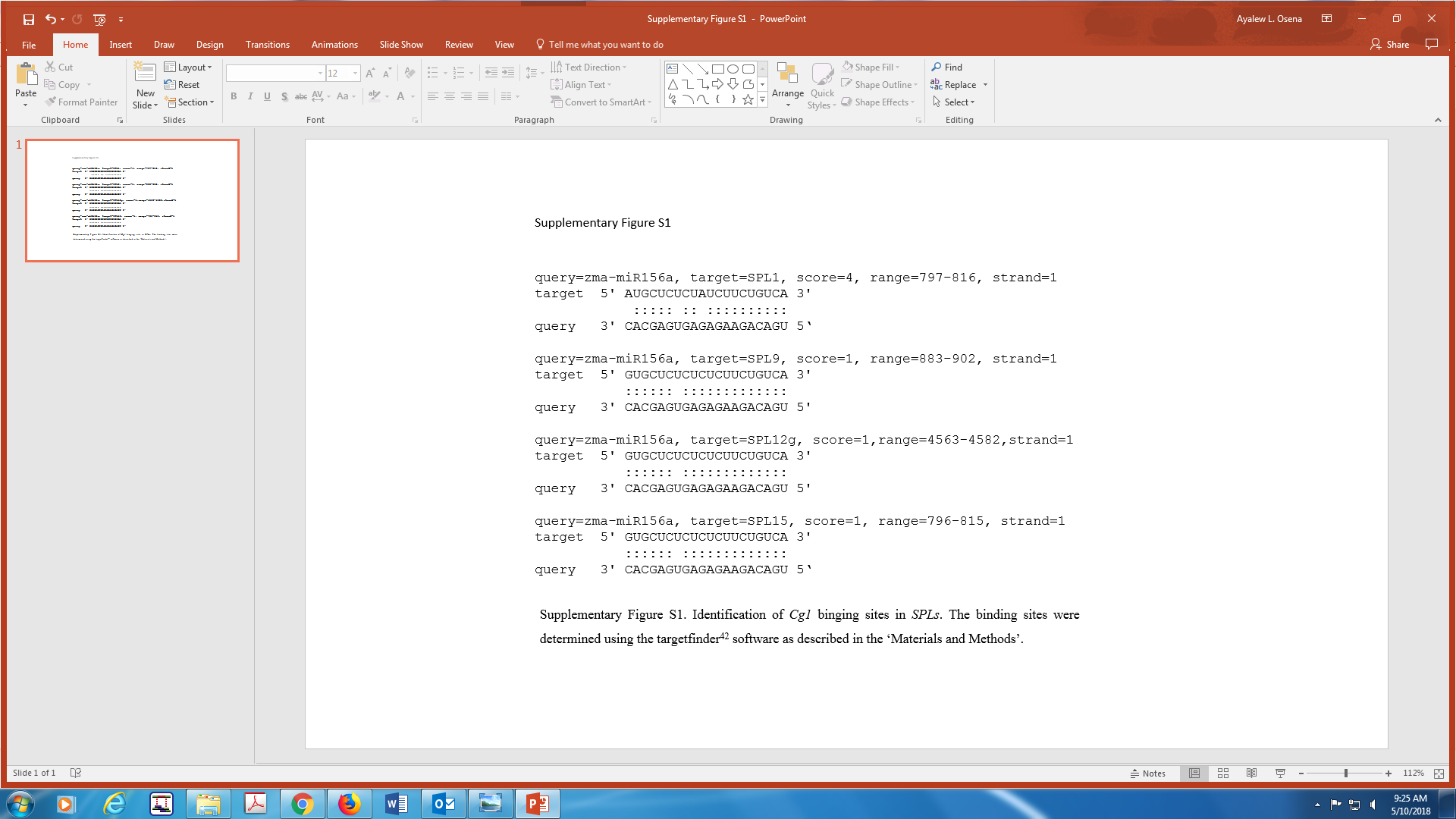

Supplement: Supplementary Materials — Supplementary Table S1: primers used for validation of transgenic lines and for gene expression analysis. Supplementary Table S2: composition of free carbohydrates prior to biomass saccharification. Supplementary Table S3: composition of free carbohydrates after biomass saccharification. Supplementary Figure S1: identification of Cg1-binding sites in SPLs. [file 8581258.f1.docx]
